# Supplementary material for: Sodium Chloroacetate Modified Polyethyleneimine/Trimesic Acid Nanofiltration Membrane to Improve Antifouling Performance
Source: Membranes (Basel). 2021 Sep 14;11(9):705. doi: 10.3390/membranes11090705 (PMC8466547; doi:10.3390/membranes11090705)
Supplement: Supplementary file 1 [file membranes-11-00705-s001.zip › membranes-1357568-supplementary.pdf]

Sodium chloroacetate modified Polyethyleneimine/Trimesic acid nanofiltration membrane to improve antifouling performance

Kaifeng Gu, Sichen Pang, Yong Zhou*, Congjie Gao

Center for Membrane and Water Science & Technology, Zhejiang University of Technology, Hangzhou 310014, China

* Corresponding Author. Email address: zhouy@zjut.edu.cn (Y.Zhou)

1. Molecular weight cut off of modified membrane.

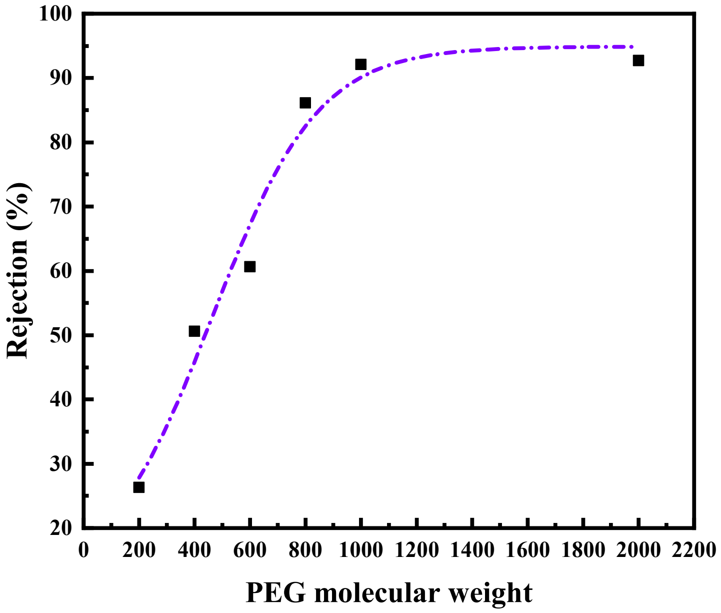

Figure S1. Molecular weight PEG removal rate curves of membrane M0-d.

The molecular weight cut-off (MWCO) of membrane M0-d was about 1000 Da (**Figure S1**). It was similar to the original membrane M0 [28]. It could be believed that the pore size of the PEI/TMA membrane had not changed significantly. The increase in hydrophilicity and the neutralization of surface chargeability could lead to the rapid transfer of water molecules.

2. XPS narrow spectrum analysis.

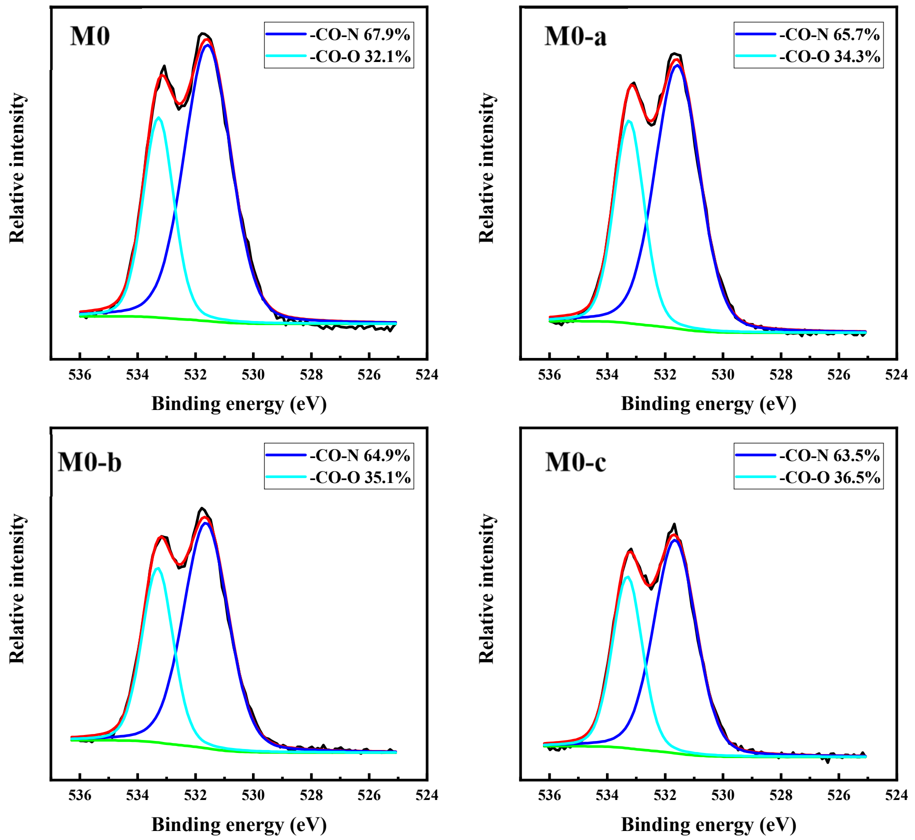

Figure S2. XPS O element narrow spectrum analysis on the surface of original membrane and modified membrane.

From the narrow spectrum peak analysis of O element, the peak of carboxyl oxygen on the surface of the membrane was obviously increased with the extension of modification time (**Figure S2**). The increased carboxyl groups only came from sodium chloroacetate. It could be believed that sodium chloroacetate was tightly adsorbed on the surface of the membrane under the action of electrostatic attraction.

3. Salt removal rate.

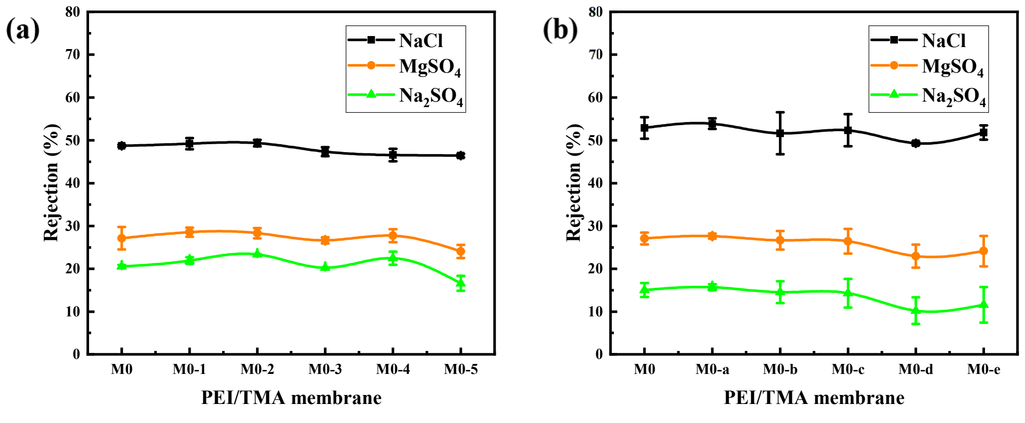

Figure S3. NaCl, $MgSO_4$, Na_2SO_4 rejection rate of modified membranes.

The PEI/TMA membrane surface was loose and positively charged, so the desalination rate of the membrane was mainly reflected in the $MgCl_2$. The removal rate of the other three salts was low, and the modification had little effect on this. The test results were shown in **Figure S3**.

4. Longer BSA test.

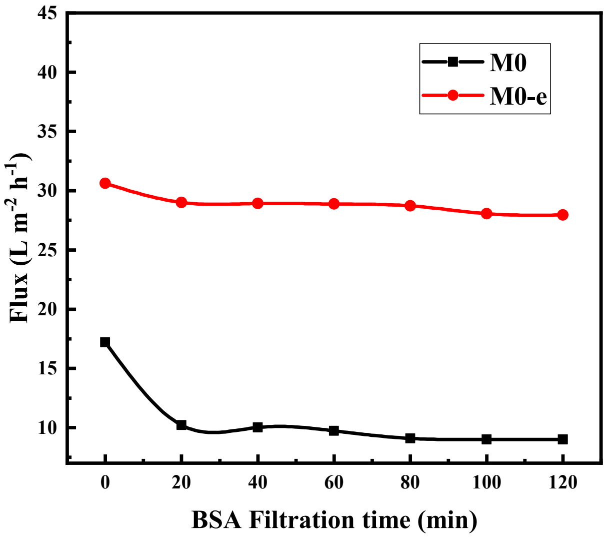

Figure S4. Anti-pollution test after 80 minutes.

It could be believed that within 20 to 40 min, the pollution had reached the limit. A longer pollution test was made and the membrane flux no longer decreased (**Figure S4**). The anti-fouling properties could be kept.

5. SEM images.

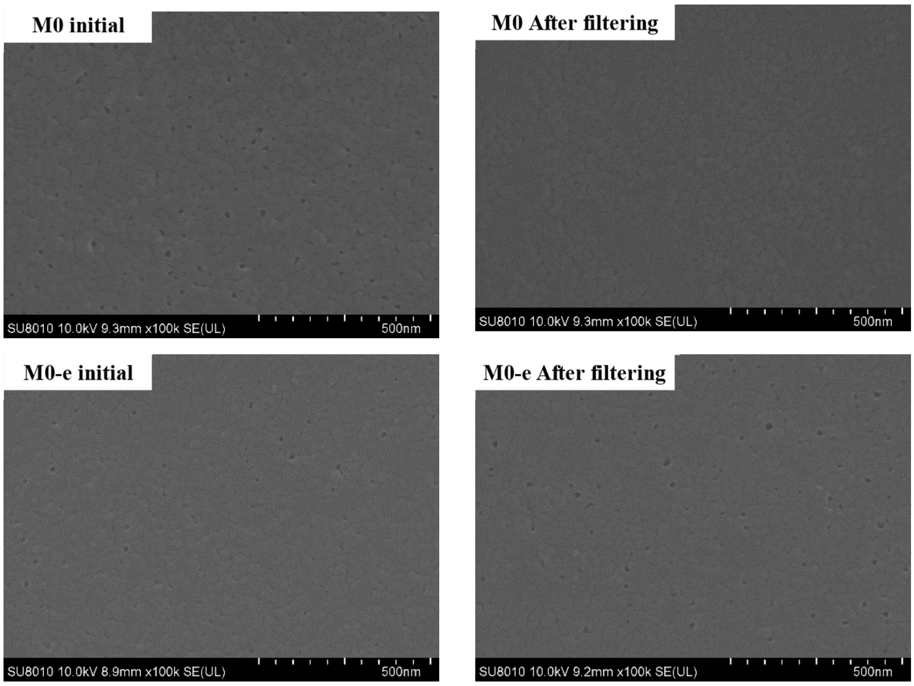

Figure S5. Comparison of M0 and M0-e before and after filtering BSA solution.

After filtering BSA, the surface of the original membrane (M0) showed partial blockage while almost no adsorption occurred on the surface of the modified membrane (M0-e).

6. Other modified membranes anti-fouling test.

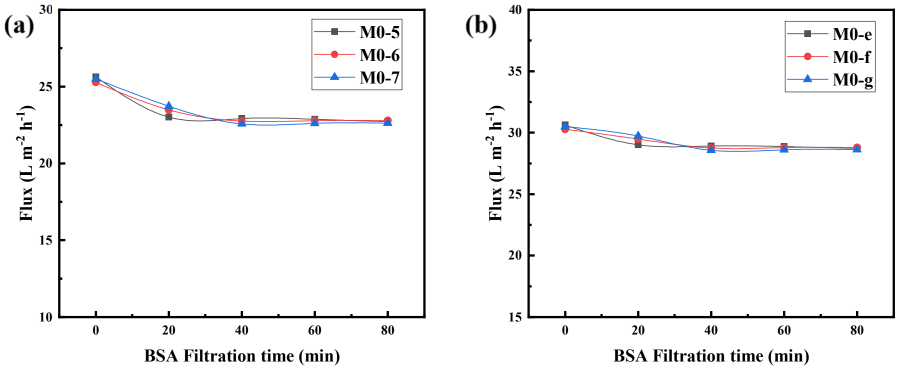

Figure S6. BSA anti-fouling test for modified membranes with higher concentration of sodium chloroacetate (**a**) and longer modification time (**b**).

The membranes modified with higher concentration of sodium chloroacetate (0.5% M0-5, 0.6% M0-6, 0.7% M0-7) and the membranes modified for longer time (10 h M0-e, 12 h M0-f, 14 h M0-g) were tested for BSA anti-pollution. The result was shown in **Figure S6**. The further modification could not improve membrane performance more effectively. It could be believed that membrane M0-e was the best modified membrane.
